# Evolution of the Kdo_2_-lipid A biosynthesis in bacteria

**DOI:** 10.1186/1471-2148-10-362

**Published:** 2010-11-24

**Authors:** Stephen O Opiyo, Rosevelt L Pardy, Hideaki Moriyama, Etsuko N Moriyama

**Affiliations:** 1 School of Biological Sciences, University of Nebraska-Lincoln, Lincoln, NE 68588-0118, USA; 2 School of Biological Sciences and Center for Plant Science Innovation, University of Nebraska-Lincoln, Lincoln, NE 68588-0118, USA; 3 Molecular and Cellular Imaging Center-South, Ohio Agricultural Research and Development Center, 2021 Coffey Road, Columbus, OH 43210, USA

## Abstract

**Background:**

Lipid A is the highly immunoreactive endotoxic center of lipopolysaccharide (LPS). It anchors the LPS into the outer membrane of most Gram-negative bacteria. Lipid A can be recognized by animal cells, triggers defense-related responses, and causes Gram-negative sepsis. The biosynthesis of Kdo_2_-lipid A, the LPS substructure, involves with nine enzymatic steps.

**Results:**

In order to elucidate the evolutionary pathway of Kdo_2_-lipid A biosynthesis, we examined the distribution of genes encoding the nine enzymes across bacteria. We found that not all Gram-negative bacteria have all nine enzymes. Some Gram-negative bacteria have no genes encoding these enzymes and others have genes only for the first four enzymes (LpxA, LpxC, LpxD, and LpxB). Among the nine enzymes, five appeared to have arisen from three independent gene duplication events. Two of such events happened within the Proteobacteria lineage, followed by functional specialization of the duplicated genes and pathway optimization in these bacteria.

**Conclusions:**

The nine-enzyme pathway, which was established based on the studies mainly in *Escherichia coli *K12, appears to be the most derived and optimized form. It is found only in *E. coli *and related Proteobacteria. Simpler and probably less efficient pathways are found in other bacterial groups, with Kdo_2_-lipid A variants as the likely end products. The Kdo_2_-lipid A biosynthetic pathway exemplifies extremely plastic evolution of bacterial genomes, especially those of Proteobacteria, and how these mainly pathogenic bacteria have adapted to their environment.

## Background

**Figure 1:**
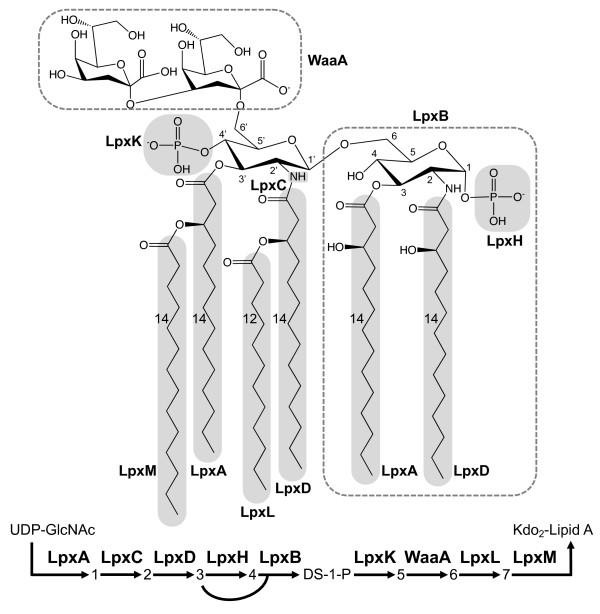
**Structure of Kdo_2_-lipid A from *E. coli *K12**. Parts joined by the nine enzymes are indicated with shadow and dashed surrounding. The nine-enzyme pathway is illustrated at the bottom. Abbreviations for enzymes and substrates are as follows: LpxA: UDP-N-acetylglucosamine acyltransferase, LpxC: UDP-3-O-(3-hydroxymyristoyl) N-acetylglucosamine deacetylase, LpxD: UDP-3-O-(3-hydroxymyristoyl) glucosamine N-acyltransferase, LpxH: UDP-2,3-diacylglucosamine pyrophosphatase, LpxB: lipid-A-disaccharide synthase, LpxK: lipid A 4'-kinase, WaaA (or KdtA): 3-deoxy-D-manno-octulosonate-lipid A transferase (or Kdo-lipid A transferase), LpxL (or HtrB): Kdo_2_-lipid IV_A _lauroyl-ACP acyltransferase, LpxM (or MsbB): Kdo_2_-lauroyl-lipid IV_A _-myristoyl-ACP acyltransferase, ACP: acyl carrier protein, UDP: uridine diphosphate, UDP-GlcNAc: UDP-N-acetylglucosamine, and DS-1-P: tetraacyldisaccharide 1-phosphate. In the pathway, substrates are shown with numbers as follows: 1: UDP-3-O-(3-hydroxytetradecanoyl)-N-acetylglucosamine, 2: UDP-3-O-(3-hydroxytetradecanoyl)-glucosamine, 3: UDP-2,3-bis(3-hydroxytetradecanoyl)-glucosamine, 4: 2,3-bis(3-hydroxytetradecanoyl)-beta-D-glucosaminyl 1-phosphate, 5: tetraacyldisaccharide 1,4'-bis-phosphate (lipid IV_A_), 6: Kdo_2_-lipid IV_A_, and 7: Kdo_2_-(lauroyl)-lipid IV_A_.

Kdo_2_-lipid A is a complex glycolipid consisting of glucosamine, 3-OH fatty acids, and unusual sugar 3-deoxy-D-*manno*-octulonsonic acid (Kdo) (Figure 1). Kdo_2_-lipid A is the principle and essential component of the outer leaflet of the outer cell wall of Gram-negative bacteria [1]. It is the membrane anchor for a wide and variable range of polysaccharide repeating units extending beyond the cell wall. Together with Kdo_2_-lipid A the polysaccharide repeating units constitute lipopolysaccharide (LPS), a class of molecules believed to occur solely in Gram-negative bacteria and Cyanobacteria and essential for their viability [2]. Lipid A is a potent immunoreactive factor responsible for triggering a macrophage mediated and frequently overwhelming immune response resulting in endotoxic shock, a potentially lethal condition. Indeed, as far as is known, all vertebrates and an assortment of invertebrates exhibit a robust and highly specific anti-LPS response.

For decades lipid A/LPS has been essentially synonymous with Gram-negative organisms, yet, despite the relative diversity of the Gram-negative bacteria, the origin and evolutionary pathway of this important molecule is virtually unknown. Presently the complex, nine-enzyme biosynthetic process of Kdo_2_-lipid A is best known from *Escherichia coli *(Figure 1). The genetic sequences for the enzymes have been derived from the *E. coli *genome (Additional file 1). This pathway and the concomitant genes in *E. coli *probably represent the most highly evolved Kdo_2_-lipid A biosynthetic pathway given the highly adapted association of *E. coli *with vertebrate enteric habitats, which depends heavily on the structure of LPS. Thus, the central question becomes: can the evolutionary radiation of LPS be described by understanding the comparative genomics of Kdo_2_-lipid A biosynthetic pathway?

Traditional classification of bacteria: *Gram-positive vs. Gram-negative*, is based on the Gram-staining procedure [3]. Gram-positive bacteria retain the crystal-violet stain, whereas Gram-negative bacteria are decolorized with alcohol or acetone and stained red with safranin or basic fuchsin. Different responses to the staining are caused by the chemical properties of the cell walls. Gram-positive bacteria have an impermeable cell wall that is made of thick peptidoglycan and is not affected by decolorization. Gram-negative bacteria, on the other hand, have a thin peptidoglycan layer and an outer membrane containing LPS, which can be disrupted by decolorization.

Phylogenetic analysis of biomolecular sequences is now considered to be more reliable for bacterial classification. Molecular phylogenies do not support the traditional simple grouping of eubacteria: Gram-negative *vs*. Gram-positive [*e.g*., [4-6]]. The most important and often used phylogenetic marker for bacteria is 16S ribosomal RNA (rRNA) due to their highly conserved nature of sequences. The second edition of Bergey's Manual of Systematic Bacteriology [7], the gold standard of bacterial classification, is mostly based on 16S rRNA phylogenies. Gram-positive bacteria are now grouped into two paraphyletic groups: the phylum Firmicutes (low G+C content Gram-positive) and the phylum Actinobacteria (high G+C content Gram-positive). Gram-negative bacteria are composed of more than 20 of highly diverged phyla. Firmicutes, a Gram-positive phylum, forms a sister cluster with a Gram-negative phylum Cyanobacteria. The phylum Proteobacteria, the largest Gram-negative bacteria group that includes *E. coli *and many pathogens, is the most derived group of bacteria.

Understanding the distribution and diversity of the Kdo_2_-lipid A biosynthetic pathway among bacteria is important for a multitude of reasons. Notwithstanding its limitation, Gram-staining is still widely used in clinical practice. It is often the first diagnostic test, which is crucial for the initial diagnostic and treatments. Kdo_2_-lipid A is the highly immunoreactive endotoxic center of LPS. The endotoxicity of LPS is dependent on and mediated by the Kdo_2_-lipid A component. Furthermore, the Kdo_2_-lipid A pathway is being considered as a target for new antibiotic development [*e.g*., [8,9]]. Kdo_2_-lipid A is required for growth of *E. coli *and most other Gram-negative bacteria. Inhibitors of the Kdo_2_-lipid A biosynthesis, therefore, can become good antibiotics against these bacteria.

In order to elucidate how the Kdo_2_-lipid A biosynthetic pathway has evolved in the bacterial kingdom, we examined the distribution of the nine enzymes involved in this pathway across 61 bacterial genomes. With Kdo_2_-lipid A as the final product, the entire pathway was expected to be highly conserved among Gram-negative bacteria. On the other hand, Gram-positive bacteria would lack some or all of the enzymes required for the Kdo_2_-lipid A biosynthesis. On the contrary, we identified a widely varied level of conservation in this pathway among Gram-negative bacteria. We showed that the currently known, considered to be "canonical", nine-enzyme pathway, which has been characterized mainly in *E. coli *and related bacteria, does not represent nor should be considered as ancestral to all Gram-negative bacteria. Rather, the nine-enzyme pathway represents the product of genomic plasticity, evolved in highly-derived Proteobacteria, especially in those closely related to *E. coli*.

## Results and discussion

### Distribution of Kdo_2_-lipid A biosynthetic enzymes across bacterial genomes

**Figure 2:**
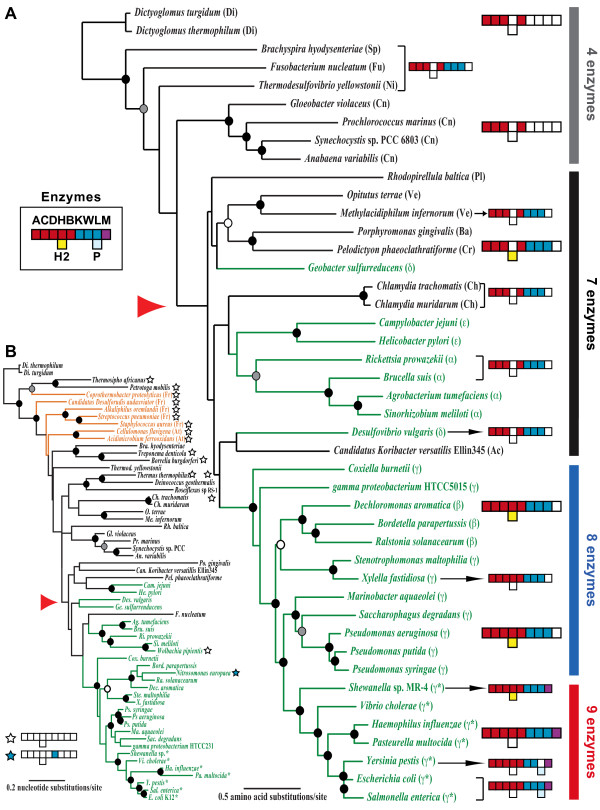
**Distribution of Kdo_2_-lipid A biosynthetic enzymes across bacteria genomes**. Maximum likelihood phylogenies were reconstructed based on concatenated six protein sequences (A) and 16S rRNA nucleotide sequences (B). Enzymes identified are color-coded as follows: LpxA (A, red), LpxC (C, red), LpxD (D, red), LpxH (H, red), LpxH2 (H2, yellow), LpxK (K, blue), WaaA (W, blue), LpxL (L, blue), LpxM (M, purple), and LpxP (P, light blue). White boxes indicate the absence of the corresponding enzymes. Bacteria that have none of the enzymes and only LpxK are marked with white and blue stars, respectively. Red arrowheads indicate the points inferred to be the emergence of the Kdo_2_-lipid A biosynthetic pathway with the last four enzymes (LpxK-LpxM). Circles at internal nodes indicate bootstrap-supporting values as follows: black circles ≥ 95%, gray circles ≥ 85%, and white circles ≥ 75%. Proteobacteria and Gram-positive bacteria are shown with green and orange letters/lines, respectively. Bacterial classifications (phylum, and for Proteobacteria, class) are shown in parentheses using the following abbreviations. Ac: Acidobacteria, Ba: Bacteroidetes, Ch: Chlamydiae, Cn: Cyanobacteria, Cr: Chlorobi, Di: Dictyoglomi, Fu: Fusobacteria, Ni: Nitrospire, Pl: Planctomycetes, Sp: Spirochaetes, Ve: Verrucomicrobia, α: Alphaproteobacteria, β: Betaproteobacteria, δ: Deltaproteobacteria, ε: Epsilonproteobacteria, and γ: Gammaproteobacteria. Group-II Gammaproteobacteria, those that have all nine enzymes, are shown with *.

Gram-negative bacteria, by definition, should have LPS-containing outer membranes; hence all these bacteria are expected to possess all genes encoding Kdo_2_-lipid A biosynthetic enzymes. These genes, on the other hand, are likely to be missing from Gram-positive bacteria unless they are used in alternative functions in these bacteria. As we expected, none of the seven Gram-positive bacteria we examined had the genes encoding these enzymes. On the other hand, we found surprisingly a wide range of presence/absence patterns with these genes among 54 Gram-negative bacteria we studied (Additional file 1). Only one group of Gammaproteobacteria including *Escherichia*, *Vibrio*, and *Shewanella *species had all nine genes required for Kdo_2_-lipid A biosynthesis. We call them Group II Gammaproteobacteria (see Figure 2A). Remaining Gammaproteobacteria (Group I; *e.g., Pseudomonas*, *Xylella fastidiosa*, *Coxiella burnetii*) as well as Betaproteobacteria *(e.g., Bordetella parapertussis *and *Dechloromonas aromatica*) had all genes except *lpxM*. All other Gram-negative bacteria are missing *lpxH *as well as *lpxM *genes. This implies that the Kdo_2_-lipid A biosynthetic pathway consisting of the nine enzymes is not ancestral, but rather a specialized, derived form found only in *E. coli *and closely related Group II Gammaproteobacteria. The LpxM protein, found only in this group, shares a sequence similarity of 47% with the LpxL protein, and appeared to be a product of gene duplication. We also found two exceptions among Proteobacteria. *Nitrosomonas europaea *(Betaproteobacteria) had only one (*lpxK*) and two species of *Walbachia *(Alphaproteobacteria) we examined had none of the nine genes. Seven other bacteria also lacked all of the nine genes although they are classified as *Gram-negative *(Figure 2B and Additional file 1).

### Gene duplication and functional specialization characterize the evolution of the Kdo_2_-lipid A biosynthetic pathway

We identified multiple gene-duplication events during the evolution of the Kdo_2_-lipid A biosynthetic pathway. *lpxA *and *lpxD *are duplicated genes and share 45% sequence similarity at the protein level. LpxA and LpxD proteins have trimer structures with different quaternary assembly and active sites [10,11]. All bacteria we examined either possess or lack both enzymes. Therefore, generation of this pair of enzymes must have been an early event in Gram-negative bacteria. Further duplications of either of the genes have happened in some lineages independently (*e.g., lpxA *duplication in *Geobacter sulfurreducens *and *lpxD *duplication in *Gloeobacter violaceus*) (see Additional file 1 for details). *lpxH *gene, which encodes pyrophosphatase [12], appeared to have arisen from a duplication of *lpxH2 *gene within the Proteobacteria lineage but before Beta- and Gammaproteobacteria divergence (Figure 2). Furthermore, *lpxL *gene seems to be prone to duplicate. We identified several such duplication events including one that generated *lpxM *in the Group II Gammaproteobacteria (Figure 2).

#### *lpxH/lpxH2 *duplication within Proteobacteria

**Figure 3:**
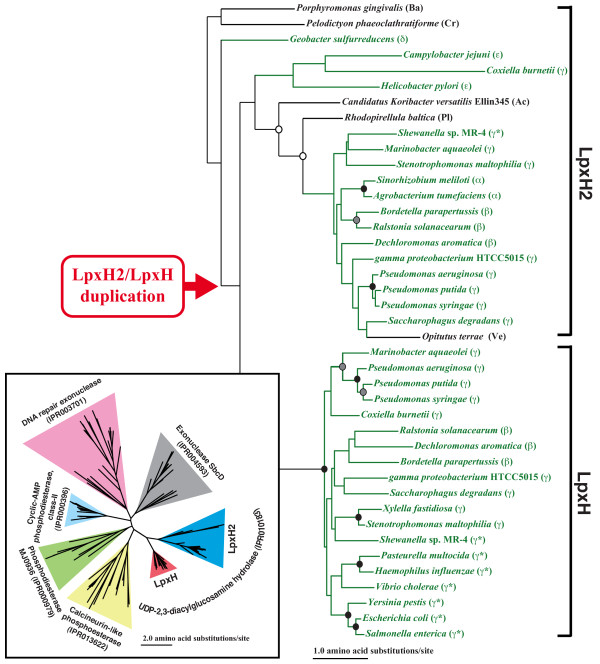
**The maximum-likelihood phylogenetic tree of LpxH, LpxH2, and related proteins**. Circles at internal nodes indicate bootstrap-supporting values as follows: black circles ≥ 95%, gray circles ≥ 85%, and white circles ≥ 70%. Proteobacteria are shown with green letters and lines. Group-II Gammaproteobacteria are indicated by *. See Figure 2 legend for abbreviations used for bacterial classification. The red arrowhead shows where the LpxH/LpxH2 duplication happened. The inset shows the maximum likelihood phylogeny reconstructed from all of the similar proteins identified in this study. It shows that each protein family (indicated with triangles with InterPro accession numbers) forms a distinct cluster.

LpxH and LpxH2 proteins share 42% sequence similarity [13]. LpxH2 candidates were found mainly in the phylum Proteobacteria, but also in some other Gram-negative bacteria (Additional file 1, Figure 2). The duplication event that created the *lpxH *gene happened within the phylum Proteobacteria before the divergence of Beta- and Gammaproteobacteria (Figures 2A and 3). Interestingly, after the duplication event, *lpxH2 *gene was lost from the Group II Gammaproteobacteria except for *Shewanella *sp. MR-4 (Figure 2A). It implies that *lpxH2 *gene is dispensable when *lpxH *gene exists. Since some of the Gram-negative bacteria that have seven enzymes in the pathway have only *lpxH2 *gene or neither *lpxH *nor *lpxH2 *genes, it is tempting to speculate that functions of LpxH and LpxH2 proteins are interchangeable or can be replaced by other non-specific phosphatases (see below for LpxI discovery). Babinski *et al*. [12] showed that *lpxH *gene from *Pseudomonas aeruginosa *compensated for the loss of *E. coli *K12 *lpxH*, but *lpxH2 *of *P. aeruginosa *did not. For those Proteobacteria that have both of LpxH and LpxH2 proteins, the function of each protein may have been optimized for their specific roles. Such specialized proteins may not be as flexible as the ancestral form.

LpxH and LpxH2 proteins belong to the metallophosphoesterase superfamily (PF00149; the Pfam database [14]). The LpxH and LpxH2 protein groups form two distinct clusters among similar bacterial proteins identified (see the inset of Figure 3). All these proteins contain a five-block motif: **D**-X_n_-**GD**-X_n_-**GNH(E/D)**-X_n_-**H**-X_n_-**GHXH**, a signature of the metallophosphoesterase superfamily. Mutational and structural analyses showed that these residues are involved in metal-ion binding and catalysis [15-18]. The five-block residues are completely conserved among all proteins we examined including LpxH and LpxH2 (shown in the inset of Figure 3) except for the third block, **GNH(E/D)**, where the histidine (H) is substituted to arginine (R) only in LpxH (Additional file 2). After the LpxH2/H ancestral protein was generated by a duplication of a gene encoding a protein similar to calcineurin phosphohydrolase (the closest relative of LpxH2/H), another duplication produced the two protein families, LpxH2 and LpxH (Figure 3). Although the function of LpxH2 has been unknown, comparisons of structural models of LpxH and LpxH2 proteins indicate that the five-block regions form similar cavities in these proteins (see Methods and Additional file 2). The histidine-to-arginine substitution in the third block in LpxH may have increased the LpxH activity significantly in Beta- and Gammaproteobacteria.

Recently, Metzger and Raetz [19] identified a gene located between *lpxA *and *lpxB *in *Caulobacter crescentus *(Alphaproteobacteria). This gene, named *lpxI*, which does not share similarity with *lpxH/lpxH2 *genes, was found to catalyze UDP-2,3-diacylglucosamine hydrolysis by a mechanism different from *lpxH*. It also rescued *lpxH*-deficient *E. coli*. *lpxI *orthologues were found in many, but not all of, bacteria that lack *lpxH *[19] (see also Additional file 1). Metzger and Raetz [19] reported that *lpxI *was found even from Gram-positive bacteria (*e.g*., Firmicutes). It suggests that replacement of *lpxH *gene function appears to be not very difficult, and multiple gene recruitments may have happened during the evolution of Kdo_2_-lipid A biosynthetic pathway.

#### Multiple *lpxL duplications *within Gram-negative bacteria

**Figure 4:**
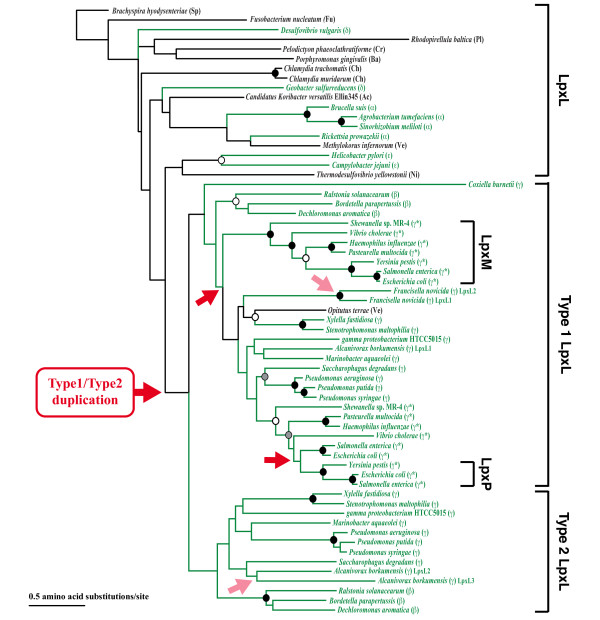
**The maximum-likelihood phylogenetic tree of LpxM and LpxL proteins**. Circles at internal nodes indicate bootstrap-supporting values as follows: black circles ≥ 95%, gray circles ≥ 85%, and white circles ≥ 70%. Proteobacteria are shown with green letters and lines. Group-II Gammaproteobacteria are indicated by *. See Figure 2 legend for abbreviations used for bacterial classification. Red arrowheads indicate where the duplication events happened. Light-red arrowheads are where the species-specific duplications are found.

Figure 4 shows the LpxL phylogeny for the entire set of bacteria we examined. Among the Gram-negative bacteria, we identified three or more independent duplication as well as loss events that involved with *lpxL*. The first duplication appears to have happened within the phylum Proteobacteria. After *Coxiella burnetti *has diverged from other Beta- and Gammabacteria, the duplication event generated two *lpxL *genes (we call them Types 1 and 2 in Figure 4). One of the duplicated *lpxL *genes, Type 2, was lost before the divergence of the Group-I Gammaproteobacteria. Interestingly this event coincided with the second gene duplication that created the *lpxM *gene in this group (Figure 4). The third duplication event is identified only in the lineage leading to closely related enterobacteria (*E. coli, Salmonella enterica*, and *Yersinia pestis*). The duplication product, LpxP, shares 74% sequence similarity with the LpxL protein. The original *lpxL *gene (Type 1) was subsequently lost from the *Y. pestis *genome (Figure 4). In addition to these major duplication events, we also identified at least two species-specific *lpxL *duplications in Gammaproteobacteria: one for Type 1 (in *Alcanivorax borkumensis *SK2; order Oceanospirillales) and another for Type 2 (in *Francisella novicida*; order Thiotrichales).

#### Functional specialization in *lpxL *and its paralogues

Although it is not clear if Types-1 and -2 LpxL function differently in all Beta/Gammabacteria, in some bacteria duplicated LpxL copies have apparently evolved to have slightly different functions. Type-1 LpxL (LpxL1) of *Bordetella pertutssis *adds secondary 2-hydroxy laurate at the position 2 of Kdo_2_-lipid A, and Type-2 LpxL (LpxL2) adds myristate at the position 2' of Kdo_2_-lipid A [20]. *Francisella novicida *has species-specific duplicated copies of Type-1 LpxL, LpxL1 and LpxL2, and they function as Kdo-dependent and -independent acyltransferases, respectively. LpxL2 of *F. novicida *can add laurate at the position 2' of lipid IV_A _without Kdo addition by WaaA, which probably accounts for a large amount of 'free lipid A' (lipid A not linked to Kdo, core sugars, and O-antigen) [21].

Acyltransferases LpxL and LpxM share 47% sequence similarity. In *E. coli*, LpxL and LpxM add laurate at the position 2' and myristate at the position 3' of Kdo_2_-lipid A, respectively [22], and have conserved catalytic dyad motifs **HX**_**4**_**D **and **HX**_**4**_**E**, respectively [23]. LpxM is not required for growth of *E. coli *K12 [24]. While *E. coli *K12 mutants for *lpxL *and *lpxM *genes can grow on minimum medium and at all temperatures, they do not grow on rich media at temperatures above 32°C [24-26]. In *Haemorphilus influenzae*, LpxL and LpxM are both myristoyl transferases [27]. Therefore, four types of related enzymes appear to have evolved after duplications: "generalist" LpxL enzyme that catalyzes the transfer of both laurate and myristate, more "specialized" LpxL enzyme that acts only either on laurate or myristate, and LpxM newly "specialized" as myristoyl transferase. LpxM is especially optimized for Group II Gammaproteobacteria, many of which live in warm body temperatures of the hosts.

#### Cold-temperature adaptation with *lpxP*

Another *lpxL *duplicate, *lpxP*, is found in *E. coli *and other closely related enterobacteria. While *E. coli *and *S. enterica *have all three paralogous genes (*lpxL*, *lpxM*, and *lpxP*), *Y. pestis *has only two derived copies (*lpxM *and *lpxP*). *lpxP *encodes the palmitoleoyl transferase, which is induced upon cold shock (12°C) [28]. In *E. coli*, palmitoleate is incorporated to Kdo_2_-lipid IV_A _by LpxP at the position where normally LpxL incorporates laurate (See Figure 1) [26]. Palmitoleoyl residue changes the properties (*e.g.*, fluidity) of the outer membrane and makes bacteria adaptable to low growth temperatures. While survival outside of an animal host is necessary in bacteria such as *E. coli*, *H. influenzae*, for example, is transmitted from animal to animal without being exposed in the colder environment. These Gram-negative bacteria do not have *lpxP *gene.

Another example of lipid-A associated adaptation is found in *Y. pestis*. *Y. pestis *changes its host from flea to mouse, cold to warm temperature environment. Due to the absence of the *lpxL *gene, *Y. pestis *produces only tetra-acylated Kdo_2_-lipid IV_A _(see Figure 1) but not hexa-acylated Kdo_2_-lipid A at 37°C (mammalian host temperature) contributing bacteria's poor Toll-like receptor 4 (TLR4) stimulating activity [29]. With *lpxP *activated, however, at 21°C (flea temperature) hexa-acylated Kdo_2_-lipid A with palmitoleate is synthesized in *Y. pestis*.

### Four-enzyme pathway in Cyanobacteria and Dictyoglomi: the primordial form?

Cyanobacteria (Cn in Figure 2) and Dictyoglomi (Di) have only four of the nine genes: *lpxA, lpxC, lpxD*, and *lpxB *(Figure 2). These genes encode the first four enzymes of Kdo_2_-lipid A biosynthesis up to the point of producing the lipid A disaccharide (DS-1-P in Figure 1), implying that this may be the primordial form of lipid A. LPS from Cyanobacteria is simpler than that of LPS of enteric bacteria and lacks Kdo, heptose, and phosphate [30-33]. It is reported that LPS from *E. coli *is more toxic than LPS from Cyanobacteria [34].

### Kdo_2_-lipid A biosynthetic pathway gene clusters

In prokaryotic genomes, many functionally related gene sets exist as gene clusters [*e.g.*, [35-37]]. As shown in Additional file 3, genes encoding some or all of the first four enzymes (*lpxA *, *lpxC*, *lpxD*, and *lpxB*) are often found in a gene cluster. Dictyoglomi and Cyanobacteria have only these four genes and all of them exist in a conserved gene cluster, *lpxD-lpxC-fabZ-lpxA-lpxB*. Note that the majority of these clusters include *fabZ*, which encodes (3R)-hydroxymyristoyl acyl carrier protein (ACP) dehydratase [38]. (3R)-hydroxymyristoyl-ACP serves as an important biosynthetic branch point. It is transferred to UDP-GlcNAc to initiate lipid A biosynthesis (Figure 1). Or it is elongated by FabZ and other enzymes of fatty acid synthesis to palmitate, which is a major component of the membrane glycerophospholipids. Therefore, this gene cluster is important for regulating the proportion of LPS and phospolipids in bacterial membranes. In some bacteria (*e.g.*, Bacteriodetes, Chorolobi, and Verrucomicrobia), the *lpxC-fabZ *part of the cluster has been fused and exists as a single gene (denoted as *lpxC/fabZ *in Additional file 3). Although *lpxH *is not found as part of these gene clusters, as mentioned before, its functional equivalent *lpxI *is found as part of the cluster (existing between *lpxA *and *B*) in Alphaproteobacteria and some other bacteria (see Additional file 3). Another often found gene cluster is *waaA-lpxK*. Again these genes encode enzymes that catalyze the two consecutive steps (see Figure 1) indicating the importance of their functional association in Kdo_2_-lipid A biosynthesis.

### Bacterial phylogeny based on Kdo_2_-lipid A biosynthetic enzymes

There have been a number of studies to reconstruct bacterial phylogenies using various molecular data [*e.g*., [6,39-41]]. Our phylogenies based on Kdo_2_-lipid A biosynthesis enzymes, analyzed concatenated (Figure 2A) or individually (Figures 3 and 4), are largely consistent with previous studies. The monophyletic cluster including both Gamma- and Betaproteobacteria is strongly supported by high bootstrap values in both Lpx protein and 16S rRNA phylogenies (both 100% in Figure 2) as well as by the shared events with *lpxH2/lpxH *and *lpxL *duplications. Group II Gammaproteobacteria, which includes the orders Enterobacteriales (*E. coli, S. enterica, Y. pestis*), Pasteurellales (*H. influenzae, Pasteurella multocida*), Vibrionales (*V. cholerae*), and Alteromonadales (*Shewanella*), also form a highly-supported cluster (100% bootstrap values in Figure 2) and all share the newly emerged *lpxM *gene. This Group II Gammaproteobacteria clustering is also supported by the phylogenetic study by Gao *et al*. [42] based on 36 protein data as well as the existence of the unique indel within the RNA polymerase β-subunit (RpoB).

Using the Dictyoglomi (Di) as the outgroup (see Method), phylogenies based on both of the Kdo_2_-lipid A biosynthetic enzymes and the 16S rRNA (Figure 2) showed that most bacteria that have no or only four enzymes (both Gram-positive and -negative bacteria) are located at basal relative to those that have seven or more of the enzymes. Exceptions include *Brachyspira hyodysenteriae *(phylum Spirochaetes) and *Thermodesulfovibrio yellowstonii *(phylum Nitrospirae). *Fusobacterium nucleatum *(phylum Fusobacteria) was also located outside of Cyanobacteria cluster in Kdo_2_-lipid A enzyme phylogeney (Figure 2A). Note, however, that none of their phylogenetic positions was supported with high bootstrap values in 16S rRNA phylogeny (Figure 2B).

### Gram-negative bacteria with no Kdo_2_-lipid A biosynthetic enzyme

Some Gram-negative bacteria (shown with stars in Figure 2B; described also in [2]) as well as Gram-positive bacteria (shown with orange letters and lines in Figure 2B) have none of the nine enzymes. As mentioned above, since both Dictyoglomi (used as the outgroup; Di) and Cyanobacteria (Cn) have the first four enzymes of the pathway, having the four-enzyme pathway appears to be the ancestral form. It implies that these enzymes must have been lost later in some groups of bacteria. Many of the Gram-negative bacteria with no Kdo_2_-lipid A biosynthetic enzyme have specialized life-styles: endosymbiomes (*e.g*., *Wolbachia *and *Borrelia burgdorferi*), obligate chemolithoautotroph (*e.g*., *Nitrosomonas europaea*), or hyperthermophiles (*e.g., Thermosipho africanus *and *Petrotoga mobilis*).

*Wolbachia *is either a parasite in arthropod hosts or a mutualist in nematode hosts. It belongs to the family Anaplasmataceae of the class Alphaproteobacteria. Bacteria in this family include *Ehrlichia chaffeensis*, *Anaplasma phagocytophilum*, and *Neorickettsia sennetsu*; all are obligatory intracellular and infect mononuclear cells and granulocytes. They have been found to lack Kdo_2_-lipid A biosynthetic genes [43,44]. Therefore, the loss of these genes must have happened in the ancestral lineage after the divergence from other Alphaproteobacteria group (see Figure 2). Lipid A is directly recognized by hosts to trigger innate-immune responses [45,46]. It is likely that the endosymbiotic bacteria have adapted to their symbiosis conditions by losing these enzymes and not producing lipid A to avoid the induction of defense response in the hosts.

*B. burgdorferi *is also an endosymbiotic bacterium that lives in ticks and transmits Lyme disease. *T. denticola *is the cause of periodontal disease. They belong to the family Spirochaetaceae (phylum Spirochaetes; order Spirochaetales). While there have been conflicting reports whether or not these bacteria have LPS [47-50], genomic analyses showed that these bacteria as well as *T. pallidum *lack Kdo_2_-lipid A biosynthetic enzymes [51-53] (see also Additional file 1). The genes encoding these enzymes have been identified from two other Spirochaetes families (Leptospiraceae and Brachyspiraceae), which diverged earlier than the family Spirochaetaceae (Figure 2A; also see [54,55]). Therefore, the gene loss event is specific to the family Spirochaetaceae.

*N. europaea *is a obligate chemolithoautotroph that derives all its energy and reductant for growth from the oxidation of ammonia to nitrite [56,57]. Only *lpxK *was detected from this Betaproteobacteria species. From the closely related *Nitrosospira multiformis *genome, only *lpxA *gene instead has been identified [58]. These ammonia-oxidizing bacteria have the smallest genomes (~3 Mb) among the Betaproteobacteria [59]. Such genome reduction is consistent with their limited lifestyles as we also described earlier for obligate endosymbionts and pathogens. Large numbers of insertion sequence elements and pseudogenes have been also found in these genomes, implying that the genomes are still undergoing reductive evolution.

### The origin of *lpx *genes found in plants

Although Kdo_2_-lipid A biosynthesis is generally considered to be a prokaryote-specific function, at least six of the nine *lpx *genes have been identified in *Arabidopsis thaliana*, *Oryza sativa*, and other plant genomes (data not shown; see also [2,60]). Furthermore, Armstrong *et al*. [61] showed that green algae and chloroplasts of garden pea were stained with affinity reagents for lipid A, indicating that these plants synthesize lipid A-like molecules. Since Cyanobacteria possess only the first four genes, all or some of the *lpx *genes in plants must have been transferred from bacteria other than Cyanobacteria.

Despite the presence of lipid A genes in plants, attempts to isolate canonical lipid A from plants using standard methods have been unsuccessful (Pardy, unpublished data); no structural data for putative lipid A in higher plants has been reported. However, as mentioned earlier, using a novel lipid A preparation technique, Snyder *et al*. [33] recently provided chemical composition and structural data for a simple lipid A from strains of a marine cyanobacteria, *Synechoccoccus*. The composition and structure of this lipid A differs significantly from that of canonical lipid A from enteric bacteria in ways hypothesized to be adaptive to marine *Synechoccoccus*. Although not only the first four genes exist in plants, lipid A in plants likely has a structure different from canonical lipid A. Further investigation is required to elucidate the origin and functions of eukaryotic *lpx *genes.

## Conclusions

Bacterial genomes are extremely plastic. Although the Kdo_2_-lipid A biosynthesis is one of the most fundamental and most conserved pathways among Gram-negative bacteria, this study showed that gene duplications as well as partial or complete losses of the genes encoding these enzymes have happened multiple times independently during bacterial evolution. Each group of bacteria took advantage of such evolutionary events to optimize the pathway and adapted to their specialized life style. The most optimized form of the pathway is found in the Proteobacteria lineage, especially among Gammaproteobacteria. The nine-enzyme pathway currently known for the Kdo_2_-lipid A biosynthesis, which is mainly studied in *E. coli *and related bacteria, is the most optimized, derived form of this pathway.

## Methods

### Bacterial genomes used

All bacterial genomes were downloaded from National Center for Biotechnology Information website [62] and other sources (see Additional file 1). Complete genomes of 61 bacterial species were sampled from 17 phyla including seven species of Gram-positive bacteria as controls. All species we examined were listed in Additional file 1. Accession numbers and sequences used in this study are available upon request.

### Searching for Kdo_2_-lipid A biosynthetic enzymes

#### BLAST similarity search

We started our similarity searches using the nine enzyme sequences obtained from the *E. coli *K12 genome as queries against the other 60 bacterial genomes (Additional file 1). For LpxI, the protein sequence from *Caulobacter crescentus *(Accession # NP_420717) was used as the query. blastp and tblastn [63] were used with a constant sample size (database length = 6,400,000; obtained as the average length of the query, 320 amino acids, multiplied by a constant genome size, 20,000). A cut-off E-value of 0.01 was used to define when there was no hit. In order to identify the orthologues, 'reciprocal' searches were performed against all genomes again using the top hit from each genome as the query.

Note that for *Brachyspira hyodysenteriae *(phylum Spirochaetes), although Bellgard *et al*. [54] mentioned that seven of the nine genes including *lpxM *was identified from the genome, we found only one copy of the LpxL protein and no other protein similar to LpxL.

#### Profile hidden Markov models (profile HMMs)

Each bacterial genome was further searched using profile HMMs. The protein sequences found by BLAST similarity searches were used to build the profile HMM for each enzyme using the w0.5 script of the Sequence Alignment and Modeling Software System (SAM, version 3.5; [64]). Since LpxH and LpxM were found only from a limited number of Proteobacteria, additional protein sequences of these enzymes (29 for LpxH, 42 for LpxM, and 47 for LpxI) were added from other bacterial species to build profile HMMs. A cut-off E-value of 0.01 and a constant sample size of 20,000 were used for profile HMM searches. To identify LpxH2 from the profile HMM search results, all sequences with the metallophosphoesterase superfamily signature **D**-X_n_-**GD**-X_n_-**GNH(E/D)**-X_n_-**H**-X_n_-**GHXH **were selected. Phylogenetic analysis (described next) was performed on the selected sequences to discriminate LpxH2 from other metallophosphoesterase. All sequences found in the cluster (78% bootstrap support) that includes proteins identified as the "UDP-2,3-diacylglucosamine hydrolase family" by InterPro (IPR010138; [65]) were selected as LpxH2 as well as LpxH (see Figure 3). Based on the phylogenetic locations, sequences belonging to LpxH and LpxH2 were decided.

### Multiple alignment and phylogenetic tree inferences

Multiple alignments of protein sequences were generated using MAFFT with the FFT-NS-i algorithm (version 6.24; [66]). The multiple alignment of 16S rRNA sequences was reconstructed using cmalign (Infernal package, version 1.02; [67]). Phylogenetic trees were reconstructed by a maximum likelihood method RAxML (version 7.0.4; [68]). The WAG and the general time reversible substitution model both with Gamma distribution for rate heterogeneity among sites were used for protein and 16S rRNA sequences, respectively. Bootstrap analysis was done with 1,000 replications for all phylogenetic reconstructions.

Many previous studies based on various molecular data showed that among the phyla included in this study, Thermotogae, Dictyoglomi, Deinococcus-Thermus, and Chloroflex have diverged earlier than other groups. Thermotogae and Dictyoglomi, for example, were shown as outmost groups based on studies from 16S and 23S rRNAs, ribosomal and other proteins, as well as based on the gene order [*e.g.*, [6,41,69,70]]. Firmicutes as an outmost group has been also supported by some other studies [40,71,72]. Our phylogenetic analysis showed paraphyly among Firmicutes. Among bacterial group that have Kdo_2_-lipid A biosynthetic enzymes, we chose Dictyoglomi as the outgroup.

For the concatenated protein phylogeny for Figure 2A, six proteins are included: LpxA, LpxC, LpxD, LpxB, WaaA, and LpxL. Each set of protein sequences were aligned individually, then concatenated as one alignment. For LpxA and LpxD, one copy was included when there was more than one duplicated copy. For LpxL, Type 1 was included when there was more than one copy.

### Structural analyses of LpxH and LpxH2 proteins

#### Conserved motifs

Figure S1A (Additional file 2) shows the five conserved blocks found in multiple alignments of the calcineurin-like phosphoesterase (PF00149), LpxH, and LpxH2 families, illustrated by sequence logos [73-75]. For PF00149, the Pfam seed alignment including 330 sequences was used. For LpxH and LpxH2, the alignments included 17 and 38 sequences, respectively, after removing sequences from closely related *Pseudomonas *species except for those from *P. aeruginosa *as representatives.

#### Structural modeling of LpxH and LpxH2 proteins

Structural modeling is performed using SWISS-MODEL Web server [76,77]. Figure S1B (Additional file 2) shows the models for *P. aeruginosa *LpxH (Pa LpxH; the positions 2- 232 out of 240 amino acids were used for modeling), *P. aeruginosa *LpxH2 (Pa LpxH2; pos. 16- 253 out of 270 aa), *Y. pestis *LpxH (Yp LpxH; pos. 3-238 out of 240 aa), and *Sinorhizobium meliloti *LpxH2 (Sm LpxH2; pos. 15-252 out of 281 aa). These four proteins were chosen to represent following different modes of LpxH/LpxH2 proteins: "LpxH2 only" mode before LpxH/LpxH2 duplication (Sm LpxH2), "dual" mode after the duplication (Pa LpxH and Pa LpxH2), and "LpxH only mode" after losing LpxH2 (Yp LpxH; modeling was not possible with the *E. coli *LpxH) (see Figure 2 for the evolution of LpxH/LpxH2 proteins). The template structure selected by the server was a potential phosphoesterase, aq_1956 of *Aquifex aeolicus *vf5 (PDB ID: 2YVT, A chain) [78]. The sequence similarities (E-values) of the four proteins against the template are as follows: 2 × 10^-06 ^(Pa LpxH), 1.7 × 10^-26 ^(Pa LpxH2), 3.70 × 10^-5 ^(Yp LpxH), and 9.20 × 10^-26 ^(Sm LpxH2). All LpxH and LpxH2 models have the root mean square deviations (RMSD) against the template structure of less than 1 Å for the colored regions and they are considered to be usable for modeling. Structural mining and graphic representation were done by PyMol [79].

#### Structural comparisons between LpxH and LpxH2

The first step of the LpxH/H2 reaction is to fix the UDP moiety (the substrate) to the enzyme. All four models show that blocks 3 and 5 form a narrow gate in the middle of the long cleft formed by blocks 1, 2, 3, and 4. The block 3 potentially plays a key role in this UDP-binding. An arginine (R), included in the block 3 of LpxH, has been found to bind directly to a uridine in a deoxyuridine triphosphate pyrophosphatase (dUTPase) [80,81]. The guanidine moiety in the arginine interacts with the uridine in UDP. Németh-Pongrácz *et al*. [82] showed that removal of the guanidine moiety almost compromised the dUTPase activity. Therefore, it is plausible that the replacement of His253 (the position number is based on the alignment shown in the sequence logo) in LpxH2 to Arg253 in LpxH has brought significant increase in the enzyme activity in LpxH. Based on the studies on the dUTPase [80] as well as on metal-dependent phosphatases [*e.g*., [15,17]], the roles of the conserved residues in LpxH and LpxH2 proteins can be inferred as follows. Negatively charged aspartic acids (D) in blocks 1, 2, and 3 form metal-binding sites (*e.g.*, for magnesium). The block 4 provides an attacking-water. Finally the block 5 holds the negatively charged sugar moiety of UDP-2,3-diacylglucosamine.

LpxH2 does not have the key arginine in the block 3. However, our modeling showed that the block 4 of Pa LpxH2 is located inside of the molecule (not visible in Figure S1B, Additional file 2) and the red-colored area is occupied by the amino acids "NRW" providing an arginine residue. Both in LpxH2 from *S. meliloti *(Sm LpxH2) and in LpxH from *Y. pestis *(Yp LpxH), the block 4's are also inside of the molecules (not visible), and other amino acids ("GDW" and "DTD", respectively) occupy the corresponding surface areas (red colored; these amino acid positions are also marked with red open boxes under the sequence logos for LpxH and LpxH2). Although the block 4 is highly conserved among LpxH and LpxH2 proteins, only the first histidine (H) is conserved among the metalophosphoesterase family (see the PF00149 sequence logo in Figure S1A, Additional file 2). Its exact spatial position may not significantly affect the enzyme function. Alternatively, a neighboring histidine may be used for the same function. After the duplication, acquiring an arginine (R) in the block 3 must have improved the enzymatic activity of LpxH (Pa LpxH and Yp LpxH). Such change did not happen in the duplicated counterpart protein, Pa LpxH2. However, a mutation to gain another arginine happened in the area structurally equivalent to the block 4. This could also increase the enzymatic activity of LpxH2 in *P. aeruginosa*.

## Authors' contributions

SOO carried out bioinformatics analysis and drafted the manuscript. HM carried out structural analysis and helped with interpretation of the data and drafting of the manuscript. RLP and ENM conceived of the study and helped with interpretation of the data and drafting of the manuscript. All authors read and approved of the manuscript.

## Supplementary Material

Additional file 1 **Distribution of Kdo_2_-lipid A biosynthetic enzymes across bacteria**. Click here for file

Additional file 2 **Structural analysis of LpxH and LpxH2 proteins**. Click here for file

Additional file 3 **Distribution of Kdo_2_-lipid A biosynthesis gene clusters**. Click here for file
