# Supplementary material for: Evolution of the Kdo2-lipid A biosynthesis in bacteria
Source: BMC Evol Biol. 2010 Nov 24;10:362. doi: 10.1186/1471-2148-10-362 (PMC3087551; doi:10.1186/1471-2148-10-362)
Supplement: Additional file 1 — Distribution of Kdo2-lipid A biosynthetic enzymes across bacteria. [file 1471-2148-10-362-S1.PDF]

**Table S1. Identification of the $Kdo_2$-lipid A biosynthetic enzymes from 61 bacterial genomes.[1]**

| Accession number[2] | Organism | LpxA | LpxC | LpxD | LpxH | LpxH2 | LpxI | LpxB | LpxK | WaaA (KdtA) | LpxL (HtrB) | LpxP | LpxM (MsbB) |
|---|---|---|---|---|---|---|---|---|---|---|---|---|---|
| **Gram-negative bacteria** | | | | | | | | | | | | | |
| **Phylum Proteobacteria; class Gammaproteobacteria (Group II)** | | | | | | | | | | | | | |
| NC_000913 | *Escherichia coli* K12 (order Enterobacteriales) | NP_414723 | NP_414638 | NP_414721 | NP_415057 | - | - | NP_414724 | NP_415435 | NP_418090 | NP_415572 | NP_416879 | NP_416369 |
| NC_012125 | *Salmonella enterica* (order Enterobacteriales) | ++ | ++ | ++ | ++ | - | - | ++ | ++ | ++ | ++ | ++ | ++ |
| NC_008150 | *Yersinia pestis* (order Enterobacteriales) | ++ | ++ | ++ | ++ | - | - | ++ | ++ | ++ | - | ++ | ++ |
| NC_002505 | *Vibrio choleae* (order Vibrionales) | ++ | ++ | ++ | ++ | - | - | ++ | ++ | ++ | ++ | - | ++ |
| NC_007146 | *Haemophilus influenzae* 86-028NP (order Pasteurellales) | ++ | ++ | ++ | ++ | - | - | ++ | ++ | ++ | ++ | - | ++ |
| NC_002663 | *Pasteurella multocida* (order Pasteurellales) | ++ | ++ | ++ | ++ | - | - | ++ | ++ | ++ | ++ | - | ++ |
| NC_008321 | *Shewanella* sp. MR04 (order Alteromonadales; family Shewanellaceae) | ++ | ++ | ++ | ++ | ++[h] | - | ++ | ++ | ++ | ++ | - | ++ |
| **Phylum Proteobacteria; class Gammaproteobacteria (Group I)** | | | | | | | | | | | | | |
| NC_002516 | *Pseudomonas aeruginosa* PAO1 (order Pseudomonadales) | ++ | ++ | ++ | ++ | ++[h] | - | ++ | ++ | ++ | 1:++<br>2:++ | - | - |
| NC_004578 | *Pseudomonas syringae* (order Pseudomonadales) | ++ | ++ | ++ | ++ | ++[h] | - | ++ | ++ | ++ | 1:++<br>2:++ | - | - |
| NC_010501 | *Pseudomonas putida* W619 (order Pseudomonadales) | ++ | ++ | ++ | ++ | ++[h] | - | ++ | ++ | ++ | 1:++<br>2:++ | - | - |
| NC_008740 | *Marinobacter aquaeolei* VT8 (order Alteromonadales; family Alteromonadaceae) | ++ | ++ | ++ | ++ | ++[h] | - | ++ | ++ | ++ | 1:++<br>2:++ | - | - |
| NC_007912 | *Saccharophagus degradans* 2-40 (order Alteromonadales) | ++ | ++ | ++ | ++ | ++[h] | - | ++ | ++ | ++ | 1:++<br>2:++ | - | - |
| NC_011071 | Stenotrophomonas maltophilia R55103 (order Xanthomonadales; family Xanthomonadaceae) | ++ | ++ | ++ | ++ | ++[h] | - | ++ | ++ | ++ | 1:++<br>2:++ | - | - |
| NC_010577 | *Xylella fastidiosa* M23 (order Xanthomonadales; family Xanthomonadaceae) | ++ | ++ | ++ | ++ | - | - | ++ | ++ | ++ | 1:++<br>2:++ | - | - |
| CMR ver. 11.0 | *Coxiella burnetii* RSA 493 (order Legionellales) | ++ | ++ | ++ | ++ | +[h] | - | ++ | ++ | ++ | +[h] | - | - |
| NZ_ABSJ00000000 | *gamma proteobacterium* HTCC5015 (unclassified) | ++ | ++ | ++ | ++ | ++[h] | - | ++ | ++ | ++ | 1:++<br>2:++ | - | - |
| **Phylum Proteobacteria; class Betaproteobacteria** | | | | | | | | | | | | | |
| NC_003295 | *Ralstonia solanacearum* GMI1000 (order Burkholderiales; family Burkholderiaceae) | ++ | ++ | ++ | ++ | ++[h] | - | ++ | ++ | ++ | 1:++<br>2:++ | - | - |
| NC_007298 | *Dechloromonas aromatica* RCB (order Rodocyclales) | ++ | ++ | ++ | ++ | ++[h] | - | ++ | ++ | ++ | 1:++<br>2:++ | - | - |
| NC_002928 | *Bordetella parapertussis* (order Burkholderiales; family Alcaligenaceae) | ++ | ++ | ++ | ++ | ++[h] | - | ++ | ++ | ++ | 1:++<br>2:++ | - | - |
| NC_004757 | *Nitrosomonas europaea* (order Nitrosomonadales) | - | - | - | - | - | - | - | ++ | - | - | - | - |
| **Phylum Proteobacteria; class Alphaproteobacteria** | | | | | | | | | | | | | |
| CMR ver. 7.0 | *Agrobacterium tumefaciens* C58 UWash (order Rhizobiales; family Rhizobiaceae) | ++ | ++ | ++ | - | ++[h] | ++ | + | ++ | ++ | ++ | - | - |
| CMR ver. 8.1 | *Brucella suis* 1330 (order Rhizobiales; family Brucellaceae) | ++ | ++ | ++ | - | - | ++ | ++ | ++ | ++ | +[h] | - | - |
| CMR ver. 1.0 | *Rickettsia prowazekii* Madrid E (order Rickettsiales; Rickettsiaceae) | ++ | ++ | ++ | - | - | ++ | ++ | ++ | ++ | ++ | - | - |

| | | | | | | | | | | | | | |
|---|---|---|---|---|---|---|---|---|---|---|---|---|---|
| CMR ver. 6.0 | *Sinorhizobium meliloti* 1021<br>(order Rhizobiales; family Rhizobiaceae) | ++ | ++ | ++ | - | ++[h] | ++ | ++ | ++ | ++ | +[h] | - | - |
| CMR ver. 10.0 | *Wolbachia pipientis* wMel<br>(order Rickettsiales; family Anaplasmataceae) | - | - | - | - | - | - | - | - | - | - | - | - |
| NC_006833 | *Wolbachia endosymbiont strain TRS of Brugia Malayi*<br>(order Rickettsiales; family Anaplasmataceae) | - | - | - | - | - | - | - | - | - | - | - | - |
| **Phylum Proteobacteria; class Epsilonproteobacteria** | | | | | | | | | | | | | |
| NC_011333 | *Helicobacter pylori*<br>(order Campylobacterales; family Helicobacteraceae) | ++ | ++ | ++ | - | +[h] | - | ++ | + | ++ | ++ | - | - |
| NC_009839 | *Campylobacter jejuni* (order Campylobacterales;<br>family Campylobacteraceae) | ++ | ++ | ++ | - | +[h] | - | ++ | + | ++ | ++ | - | - |
| **Phylum Proteobacteria; class Deltaproteobacteria** | | | | | - | | | | | | | | |
| NC_002939 | *Geobacter sulfurreducens* (order Desulfuromonadales) | 1:++<br>2:++ | ++ | ++ | - | +[h] | - | ++ | ++ | ++ | ++ | - | - |
| NC_002937 | *Desulfovibrio vulgaris* (order Desulfovibriales) | ++ | ++ | ++ | - | - | - | ++ | ++ | ++ | ++ | - | - |
| | | | | | | | | - | | | | | |
| **Phylum Acidobacteria** | | | | | | | | | | | | | |
| NC_008009 | *Candidatus Koribacter versatilis* Ellin345 | ++ | ++ | 1:++<br>2:++ | - | +[h] | - | ++ | ++ | ++ | + | - | - |
| **Phylum Bacteroidetes** | | | | | | | | | | | | | |
| CMR ver. 3.1 | *Porphyromonas gingivalis* W83 | ++ | ++ | ++ | - | +[h] | - | ++ | ++ | ++ | +[h] | - | - |
| **Phylum Chlamydiae** | | | | | | | | | | | | | |
| CMR ver. 18.0 | *Chlamydia trachomatis* A/HAR013 | ++ | ++ | ++ | - | - | - | ++ | ++ | ++ | ++ | - | - |
| NC_002620 | *Chlamydia muridarum Nigg* | ++ | ++ | ++ | - | - | - | ++ | ++ | ++ | ++ | - | - |
| **Phylum Verrucomicrobia** | | | | | | | | | | | | | |
| NC_010794 | *Methylacidiphilum infernorum V4*<br>(unclassified Verrucomicrobia) | 1:++<br>2:++ | ++ | ++ | - | - | +[h] | ++ | ++ | ++ | +[h] | - | - |
| NC_010571 | *Opitutus terrae* PB9001 (class Opitutae) | 1:++<br>2:++ | ++ | ++ | - | +[h] | ++ | ++ | ++ | ++ | ++ | - | - |
| **Phylum Planctomycetes** | | | | | | | | | | | | | |
| NC_005027 | *Rhodopirellula baltica* SH 1 | ++ | ++ | ++ | - | +[h] | ++ | ++ | ++ | ++ | +[h] | - | - |
| **Phylum Cyanobacteria** | | | | | | | | | | | | | |
| NC_007413 | *Anabaena variabilis* ATCC 29413 (order Nostocales) | ++ | ++ | ++ | - | - | - | ++ | - | - | - | - | - |
| CyanoBase | *Synechocystis* sp. PCC 6803 (order Chroococcales) | ++ | ++ | ++ | - | - | - | ++ | - | - | - | - | - |
| NC_008820 | *Prochlorococcus marinus* MIT 9303 (order Prochlorales) | ++ | ++ | ++ | - | - | - | ++ | - | - | - | - | - |
| NC_005125 | *Gloeobacter violaceus*<br>(class Gloeobacteria; order Gloeobacterales) | ++ | ++ | 1:++<br>2:++<br>3:++ | - | - | - | ++ | - | - | - | - | - |
| **Phylum Fusobacteria** | | | | | | | | | | | | | |
| NC_003454 | *Fusobacterium nucleatum subsp. nucleatum* | ++ | ++ | ++ | - | - | +[h] | ++ | ++ | ++ | ++[h] | - | - |
| **Phylum Chlorobi** | | | | | | | | | | | | | |
| NC_011060 | *Pelodictyon phaeoclathratiforme* BU-1 | ++ | ++ | ++ | - | +[h] | - | ++ | ++ | ++ | + | - | - |
| **Phylum Nitrospirae** | | | | | | | | | | | | | |
| NC_011296 | *Thermodesulfovibrio yellowstonii* | ++ | ++ | ++ | - | - | ++ | ++ | ++ | ++ | ++ | - | - |
| **Phylum Deinococcus-Thermus** | | | | | | | | | | | | | |
| NC_008025 | *Deinococcus geothermalis* DSM 11300 | - | - | - | - | - | - | - | - | - | - | - | - |

| | | | | | | | | | | | | | |
|---|---|---|---|---|---|---|---|---|---|---|---|---|---|
| | (class Deinococci; order Deinococcales) | | | | | | | | | | | | |
| NC_005838 | *Thermus thermophilus* HB27 | - | - | - | - | - | - | - | - | - | - | - | - |
| | (class Deinococci; order Thermales) | | | | | | | | | | | | |
| **Phylum Chloroflexi** | | | | | | | | | | | | | |
| NC_009523 | *Roseiflexus* sp. RS01 | - | - | - | - | - | - | - | - | - | - | - | - |
| **Phylum Spirochaetes** | | | | | | | | | | | | | |
| NC_012225 | *Brachyspira hyodysenteriae* WA1 | ++ | ++ | ++ | - | - | - | ++ | ++ | ++ | + | - | - |
| | (order Spirochaetales; family Brachyspiracea) | | | | | | | | | | | | |
| NC_012197 | *Borrelia burgdorferi* WI91023 | - | - | - | - | - | - | - | - | - | - | - | - |
| | (order Spirochaetales; family Spirochaetaceae) | | | | | | | | | | | | |
| NC_002967 | *Treponema denticola* ATCC 35405 | - | - | - | - | - | - | - | - | - | - | - | - |
| | (order Spirochaetales; family Spirochaetaceae) | | | | | | | | | | | | |
| **Phylum Thermotogae** | | | | | | | | | | | | | |
| NC_010003 | *Petrotoga mobilis* SJ95 | - | - | - | - | - | - | - | - | - | - | - | - |
| NC_011653 | *Thermosipho africanus* TCF52B | - | - | - | - | - | - | - | - | - | - | - | - |
| **Phylum Dictyoglomi** | | | | | | | | | | | | | |
| NC_011297 | *Dictyoglomus thermophilum* H-6-12 | ++ | ++ | ++ | - | - | - | ++ | - | - | - | - | - |
| NC_011661 | *Dictyoglomus turgidum* DSM 6724 | ++ | ++ | ++ | - | - | - | ++ | - | - | - | - | - |
| **Gram-positive bacteria** | | | | | | | | | | | | | |
| **Phylum Firmicutes; class Clostridia** | | | | | | | | | | | | | |
| NC_011295 | *Coprothermobacter proteolyticus* DSM 5265 | - | - | - | - | - | - | - | - | - | - | - | - |
| | (order Thermoanaerobacterales) | | | | | | | | | | | | |
| NC_010424 | *Candidatus Desulforudis audaxviator* MP104C | - | - | - | - | - | - | - | - | - | - | - | - |
| | (order Clostridiales; family Peptococcaceae) | | | | | | | | | | | | |
| NC_009922 | *Alkaliphilus oremlandii* OhILAs | - | - | - | - | - | - | - | - | - | - | - | - |
| | (order Clostridiales; family Clostridiaceae) | | | | | | | | | | | | |
| **Phylum Firmicutes; class Bacilli** | | | | | | | | | | | | | |
| NC_009782 | *Staphylococcus aureus* Mu3 (order Bacillales) | - | - | - | - | - | - | - | - | - | - | - | - |
| NC_010582 | *Streptococcus pneumoniae* CGSP14 | - | - | - | - | - | - | - | - | - | - | - | - |
| | (order Lactobacillales) | | | | | | | | | | | | |
| **Phylum Actinobacteria; class Acidimicrobidae** | | | | | | | | | | | | | |
| NC_013124 | *Acidimicrobium ferrooxidans* (order Acidimicrobiales) | - | - | - | - | - | - | - | - | - | - | - | - |
| **Phylum Actinobacteria; class Actinobacteridae** | | | | | | | | | | | | | |
| NC_014151 | *Cellulomonas flavigena* (order Actinomycetales) | - | - | - | - | - | - | - | - | - | - | - | - |

[1]Kdo$_2$-lipid A biosynthetic enzymes were searched using *E. coli* protein sequences (shown with accession numbers) with BLASTP as well as TBLASTN similarity search. Orthologous sequences were identified based on the results of reciprocal searches. For LpxI, the protein sequence from *Caulobacter crescentus* (Accession # NP_420717) was used as the query. See Materials and Methods section of the main text for more details. Existence or absence of orthologous genes are shown as follows: '+' for $10^{-8}$ <= E-value <= 0.01, '++' for E-value < $10^{-8}$, or '-' for no hit (E-value > 0.01). For those identified with profile hidden Markov models are marked with 'h'.

[2]Accession numbers are from National Center for Biotechnology Information (NCBI; http://www.ncbi.nlm.nih.gov/) when it is not specified otherwise. Other data sources are Comprehensive Microbial Resource (CMR; http://cmr.jcvi.org/tigr-scripts/CMR/CmrHomePage.cgi) and CyanoBase (ftp://ftp.kazusa.or.jp/pub/CyanoBase/Synechocystis).
