# Supplementary material for: Evolution of the Kdo2-lipid A biosynthesis in bacteria
Source: BMC Evol Biol. 2010 Nov 24;10:362. doi: 10.1186/1471-2148-10-362 (PMC3087551; doi:10.1186/1471-2148-10-362)
Supplement: Additional file 2 — Structural analysis of LpxH and LpxH2 proteins. [file 1471-2148-10-362-S2.PDF]

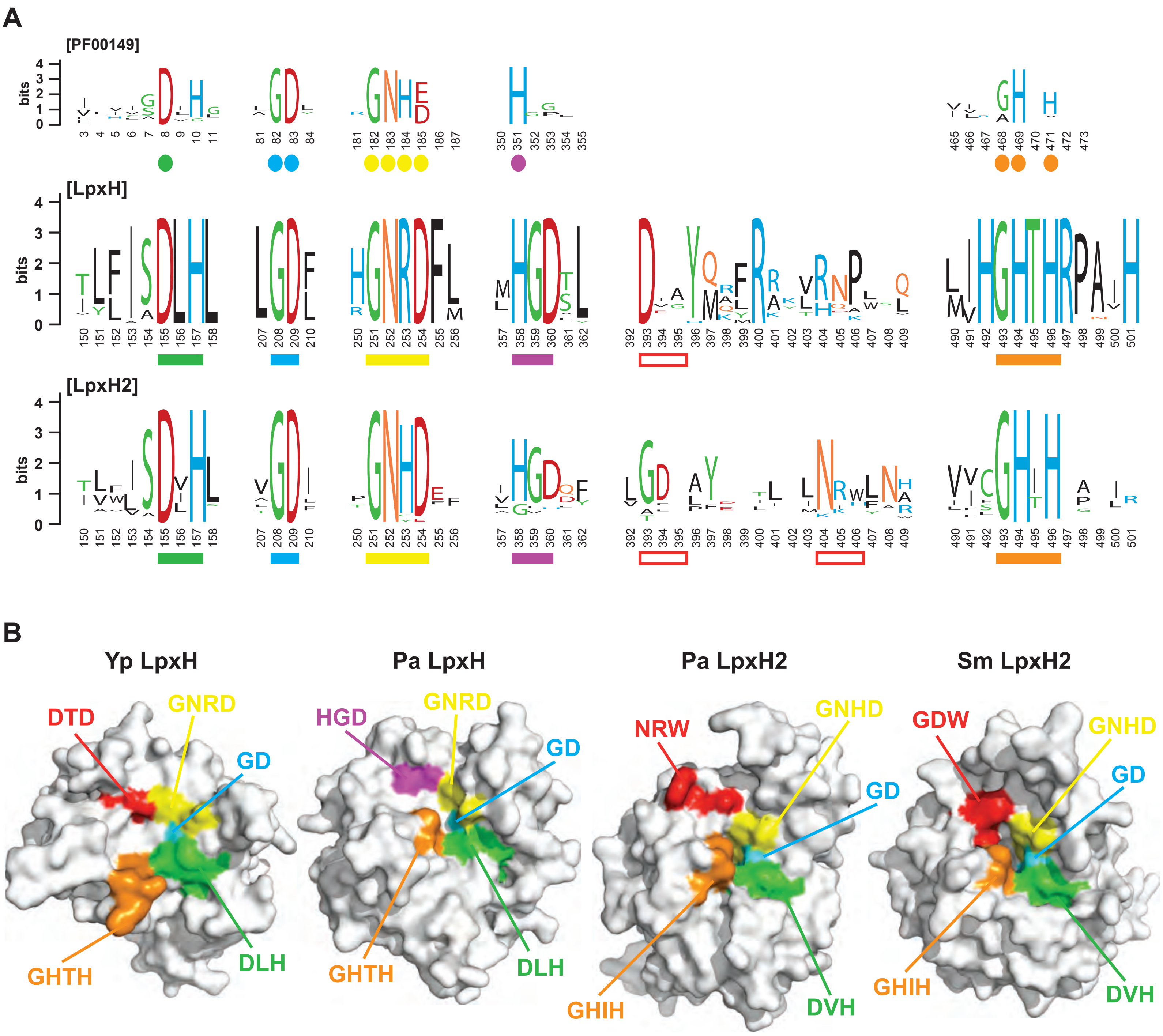

Figure S1. Structural analysis of LpxH and LpxH2 proteins. A. The five conserved blocks identified for the calcineurin-like phosphoesterase (PF00149), LpxH, and LpxH2 families. The amino acid positions for the five-block motif **D-X$_n$-GD-X$_n$-GNH(E/D)-X$_n$-H-X$_n$-GHXH** are marked with dots under the PF00149 sequence logo. Under the LpxH and LpxH2 sequence logos, the regions corresponding to the five blocks are marked with colored filled boxes. **B. Structural modeling of LpxH and LpxH2 proteins.** Residues for the five conserved blocks are highlighted with different colors: green (block 1), sky blue (block 2), yellow (block 3), magenta (block 4), and orange (block 5). These colors correspond to those used to mark the sequence logos (filled boxes) in the panel A.
