# Supplementary material for: Evolution of the Kdo2-lipid A biosynthesis in bacteria
Source: BMC Evol Biol. 2010 Nov 24;10:362. doi: 10.1186/1471-2148-10-362 (PMC3087551; doi:10.1186/1471-2148-10-362)
Supplement: Additional file 3 — Distribution of Kdo2-lipid A biosynthesis gene clusters. [file 1471-2148-10-362-S3.PDF]

Table S2. Distribution of Kdo$_2$-lipid A biosynthesis gene clusters.[1]

Bacterial groups	*lpxD-fabZ-lpxA-lpxB* cluster	Other gene cluster
Dictyoglomi	***lpxD-lpxC***-*fabZ*-***lpxA-lpxB***	
Spirochaetes	*fabZ*-***lpxA-lpxB***	
Fusobacteria	***lpxC***-*fabZ*-***lpxA-lpxI-lpxB***	
Nitrospirae	***lpxD***-*fabZ*-***lpxA-lpxI***	***lpxB-waaA***
Cyanobacteria	***lpxD-lpxC***-*fabZ*-***lpxA-lpxB*** or ***lpxC***-*fabZ*-***lpxA-lpxB***<br>(*Synechocystis* sp.: no cluster)	
Planctomycetes	***lpxC-lpxA, lpxD-lpxI***	
Verrucomicrobia	***lpxC***/*fabZ*-***lpxA***	
Bacteroidetes	***lpxD-lpxC***/*fabZ*-***lpxA***	
Chlorobi	***lpxC***/*fabZ*	
Chlamydiae	***lpxC***-*fabZ*-***lpxA***	(*C. muridarum*: ***waaA-lpxK***)
Acidobacteria	*fabZ*-***lpxA, lpxL-lpxD****	***waaA-lpxK***
[Proteobacteria]		
Epsilonproteobacteria	*fabZ*-***lpxA***	
Deltaproteobacteria	*G. sulfurreducens*: ***lpxD***-*fabZ*-***lpxA***-X-X-***lpxB***-X-X-***lpxK-waaA***<br>*D. vulgaris*: ***lpxD***-*fabZ*-***lpxA-lpxI***	
Alphaproteobacteria	***lpxD***-*fabZ*-***lpxA-lpxI-lpxB*** (*R. prowazekii*: ***lpxD***-*fabZ*-***lpxA***)	***waaA***-X-***lpxK*** (*R. prowazekii*: ***lpxK-lpxL***)
Betaproteobacteria	***lpxD***-*fabZ*-***lpxA-lpxB***	***lpxL1-lpxL2***
Gammaproteobacteria (Group II)	***lpxD***-*fabZ*-***lpxA-lpxB*** (*H. influenzae*: *fabZ*-***lpxA-lpxB***)	(*V. cholerae:* ***lpxL-lpxM***)
Gammaproteobacteria (Group I)	***lpxD***-*fabZ*-***lpxA-lpxB*** (*C. bunetii*: ***lpxD***-*fabZ*-***lpxA***)<br>(gamma proteobacterium‡: ***lpxD***-*fabZ*-***lpxA***-X-X-***lpxB***†)	(*M. aquaeolei, S. maltophilia, X. fastidiosa*: ***lpxL-waaA***†)<br>(gamma proteobacterium‡: ***lpxL-waaA***)

[1]Gene clusters were identified from the 61 bacterial genomes listed in Table S1. The genes encoding the nine enzymes are shown in boldface. 'X' indicates any one gene located within the gene cluster. '*lpxC/fabZ*' denotes a fused gene. All genes are ordered from 5' to 3' direction.

*One of the two duplicated *lpxD* genes.

†The gene is located on the different strand.

‡The *gamma proteobacterium* HTCC5015 genome is not completely assembled.
